# Depletion of myeloid-derived suppressor cells alleviates kidney damage in murine membranous nephropathy

**DOI:** 10.3389/fimmu.2025.1623613

**Published:** 2025-08-27

**Authors:** Huimin Li, Anhui Wei, Zhanchuan Ma, Li Yang, Xia Xiao, Chang Liu, Chunyan Teng

**Affiliations:** ^1^ Department of Clinical Laboratory, The Second Hospital of Jilin University, Changchun, Jilin, China; ^2^ Institute of Frontier Medical Sciences, Jilin University, Changchun, China; ^3^ Central Laboratory, The First Hospital of Jilin University, Changchun, Jilin, China; ^4^ Institute of Zoonosis, and College of Veterinary Medicine, Jilin University, Changchun, China

**Keywords:** murine primary membranous nephropathy model, myeloid-derived suppressor cells, T helper 2, T helper 17, regulatory T

## Abstract

Based on previous studies on myeloid-derived suppressor cells (MDSCs) and T helper 17 (Th17) cells in patients with primary membranous nephropathy (PMN), we successfully established a murine PMN model to investigate the relationship between MDSCs, T cells, and disease progression. Our study demonstrated that MDSCs and their subclasses, as well as Th17 and T helper 2 (Th2) immune responses, were enhanced. In contrast, the proportion of T helper 1 (Th1) and regulatory T (Treg) cells decreased with PMN progression. Depletion of MDSCs with gemcitabine reduced the proportion of Th17 and Th2 cells and the expression of related transcription factors. Conversely, the proportions of Th1 and Treg cells increased in the circulation, spleen, lymph nodes, and kidneys, alleviating the clinical manifestations and pathological damage to the renal tissue in PMN model mice. These findings suggest that MDSCs, along with Th17 and Th2 responses, play critical roles in PMN progression. MDSCs may contribute to disease progression by regulating the differentiation and immune response of T-cell subclasses. The data provide new insights into the etiology, pathogenesis, clinical diagnosis, and treatment of membranous nephropathy.

## 1 Introduction

Primary membranous nephropathy (PMN) is a kidney-specific autoimmune glomerular disease and is the most common cause of idiopathic nephrotic syndrome in adults worldwide (1–6). Approximately 85% of PMN cases are mediated by antibodies targeting the M-type phospholipase A2 receptor (anti-PLA2R), 3–5% are associated with thrombospondin type 1 domain-containing 7A, and the remaining 10% result from unidentified mechanisms (7–9).

Recent studies using laser microdissection and mass spectrometry have identified novel antigens associated with PMN, including exotosin 1 and exotosin 2, neural EGF-like-1 protein, semaphorin 3B, and protocadherin 7 (10). Despite these advances, a comprehensive understanding of PMN pathogenesis, including disease triggers, immunoglobulin G (IgG) subclass participation, complement system pathways, and the roles of other immune mediators such as T helper (Th) cells and Tregs (6), remains incomplete. Current concepts are largely derived from earlier studies conducted in the Heymann MN rat model (11). The development of novel targeted therapies has been hindered by the lack of a robust mouse model of PMN that would provide a detailed understanding of the pathogenic mechanisms.

Myeloid-derived suppressor cells (MDSCs) are a heterogeneous population of bone marrow-derived myeloid progenitors that fail to differentiate into mature myeloid cells (12, 13). They have been implicated in autoimmune diseases involving Th17 cells, including systemic lupus erythematosus (SLE) (14), rheumatoid arthritis (RA) (15, 16), and experimental autoimmune encephalitis (EAE) (17). In addition, MDSCs play a role in Th2-mediated autoimmune diseases and various human and murine tumors (18–20). Reports on the interaction between MDSCs, Th17 cells, and Th2 cells in different autoimmune diseases and at different disease stages are conflicting.

Our previous study showed a significant increase in MDSCs in peripheral blood mononuclear cells (PBMCs) of patients with PMN. Enhanced Th2 and Th17 immune responses correlated positively with disease activity in patients with PMN. In contrast, the Th1 response was weakened, and the Treg ratio was downregulated. We concluded that MDSCs likely contribute to PMN pathogenesis and progression by enhancing the Th17 response (21). The use of animal models for *in vivo* experimental studies is essential to elucidate further the relationship between MDSCs and T cell subclasses in PMN pathogenesis, disease progression, monitoring, therapeutic strategies, and preclinical experimental therapies. Here, we constructed a murine PMN model using recombinant human type IV collagen α3 chain non-collagenous region 1 domain (rh-α3NC1) (11). We observed increased proportions of MDSCs and their subclasses in the peripheral blood and spleens of PMN mice. After MDSC depletion with gemcitabine in a murine PMN model, renal tissue damage was alleviated, Th17 and Th2 immune responses and associated transcription factors were reduced, and the Th1 immune response and Treg ratio were enhanced.

## 2 Materials and methods

### 2.1 Materials

Rh-α3NC1 and gemcitabine were purchased from Creative BioMart (New York, USA) and Sigma (Staint Louis, Missouri, USA), respectively.

### 2.2 Animal experiments

Six-to ten-week-old female and male DBA/1 mice were obtained from the Beijing Vital River Laboratory Animal Technology (Beijing, China). All mice were housed under specific pathogen-free conditions with a 12-h light/dark cycle at 22 °C at the animal facility of the Institute of Translational Medicine, Jilin University. All animal experiments were conducted (No. 20210826) according to the institutional guidelines of the Institute for Laboratory Animal Research at the First Hospital of Jilin University Laboratory Animal Center (Changchun, China).

Groups of four–six female or male mice were immunized subcutaneously at two sites on the back with rh-α3NC1 (30 mg in 50 mL phosphate buffered saline (PBS) emulsified in an equal volume of Complete Freund’s Adjuvant (Sigma, Saint Louis, Missouri, USA)), followed by a booster immunization 3 weeks later using the same antigen dose in Incomplete Freund’s Adjuvant (Sigma, Saint Louis, Missouri, USA). Control mice (Complete Freund’s Adjuvant, CFA) received PBS instead of antigens. Blood samples were collected from the jugular vein every 3 or every 6 weeks;; spot urine samples were collected every two weeks using a urine collection station. The mice were regularly monitored for signs of disease and euthanized if they developed edema, ascites, abnormally high blood urea nitrogen levels, excessive body weight loss (> 10% per week), or lethargy. The remaining mice were sacrificed at ~18 weeks post-immunization, and blood, kidneys, spleen, and lymph nodes were collected for further analyses. Three independent experiments were performed for each strain.

### 2.3 MDSC depletion

Some antigen-immunized mice (Ag model mice) were injected *intraperitoneally* with 100 mg/kg gemcitabine (Sigma-Aldrich) diluted in PBS on days 4, 7, 10, and 13 after the second immunization, following established protocols (17, 22). MDSC depletion after gemcitabine treatment was assessed by flow cytometry analysis of peripheral blood samples after staining.

### 2.4 Evaluation of kidney function and renal histopathology

The urinary albumin-to-creatinine ratio (ACR) was measured in spot urine samples by ELISA using a mouse albumin-to-creatinine ratio assay kit (Abcam, Montgomery, TX, USA). Urea levels were determined using a colorimetric urea assay kit (Abcam, Montgomery, TX, USA) following the manufacturer’s instructions.

Renal tissue samples from mice with PMN were fixed in 10% buffered formalin, dehydrated through graded ethanol, embedded in paraffin, and sectioned (2-μm thick) for histological staining with hematoxylin and eosin, periodic acid-Schiff, periodic acid-silver methenamine, and Masson’s trichrome stains. A minimum of 50 glomeruli from each mouse were evaluated for mesangial proliferation, global or segmental sclerosis, spike formation, necrosis, and crescent formation. IgG levels in frozen kidney sections were determined by immunofluorescence staining with goat anti-mouse IgG-FITC (Absin, Glostrup, Denmark). For transmission electron microscopy (TEM), kidney cortex samples were fixed in 4% paraformaldehyde in 0.1 M cacodylate buffer (pH 7.4), post-fixed in 1.25% aqueous osmium tetroxide, dehydrated through an ethanol series, embedded in plastic, sectioned using a diamond knife, and stained with 4% uranyl acetate and lead citrate. All the histological and ultrastructural assessments were performed in a blinded manner.

### 2.5 Flow cytometric analysis

Flow cytometry was used to determine the phenotype of the mouse MDSCs and T cells using various combinations of fluorochrome-conjugated monoclonal antibodies (mAbs): anti-mouse-CD11b APC/cy7 (M1/70), Gr-1 PerCP-Cy5.5 (RB6-8C5), Ly6C-PE/CY7(AL-21), Ly6G-FITC (RB6-8C5) from BD biosciences; anti-mouse-CD4 FITC (GK1.5), IL-17A APC(TC11-18H10.1), interferon (IFN)-γ PerCP/Cyanine5.5 (XMG1.2), IL-13 PE (W17010B), and FoxP3 PE (150D) from BioLegend; Isotype controls included mouse IgG1 (RMG1-1, MOPC-21), IgG2b (MPC-11), IgM (MM-30), IgG1 (MOPC-21) and IgG2a (R19-15) from BioLegend. For intracellular staining, the cells were first stained for surface antigens, fixed, permeabilized with intracellular fixation and permeabilization buffer (eBiosciences), and stained with fluorochrome-conjugated monoclonal antibodies against relevant intracellular proteins. All samples were processed on a fluorescence-activated cell sorter (LSR Fortessa, Lakes, USA) and analyzed using FlowJo software. The gating strategy for flow cytometry detecting MDSCs, Th17/Th2/Th1 and Treg cells were shown in **Supplementary Figure S5**.

### 2.6 Quantitative real-time PCR

Total RNA was extracted using AxyPrepTM Multisource Total RNA Miniprep Kit, and cDNA was synthesized using SuperScript II Reverse Transcriptase. All PCRs were performed in triplicate on an ABI StepOnePlus system using TransScript Green Two-Step Quantitative Real-Time PCR SuperMix. β-Actin served as an internal control for normalization by standard 2^−ΔΔCT^ calculation as described previously (23). The primer sequences used were as follows: mouse actin, 5′-TTCACACCCCAGCCATG-3′(forward) and 5′- CCTCGTAGATGGGCACAGT-3′(reverse); mouse *IL-17A*, 5′- GGCCCTCAGACTACCTCAC -3′(forward) and 5′- TCTCGACCCTGAAGTGAGG -3′(reverse); mouse *RORα*, 5′-CTACATTGACGGGCACACC-3′(forward) and 5′-ACACAGTTGGGGAAGTCTCG -3′(reverse); mouse *RORγt*, 5′- GACCCACACCTCACAAATTGA -3′(forward) and 5′- AGTAGGCCACATTACACTGCT-3′(reverse); mouse *IL-13*, 5′- GAATCCAGGGCTACACAGAAC-3′(forward) and 5′- AACATCACACAAGACCAGACT-3′(reverse); and mouse *GATA3*, 5′- GAAGGCATCCAGACCCGAAAC -3′(forward) and 5′- ACCCATGGCGGTGACCATGC-3′(reverse).

### 2.7 Statistical Analyses

Data are presented as the mean ± standard deviation (SD) of the number of independent replicates indicated in the corresponding figure legends. Statistical analyses were performed using GraphPad Prism (version 9.2.0). For comparisons between two groups, statistical significance was determined using the nonparametric Mann-Whitney U test. For comparisons among more than two groups, statistical significance was assessed using one-way analysis of variance (ANOVA) followed by Tukey’s *post hoc* test for multiple comparisons. Statistical significance was set at *P* < 0.05 for all tests.

## 3 Results

### 3.1 Expansion of MDSCs in peripheral blood correlates with disease development in the PMN model mice

**Figure 1:**
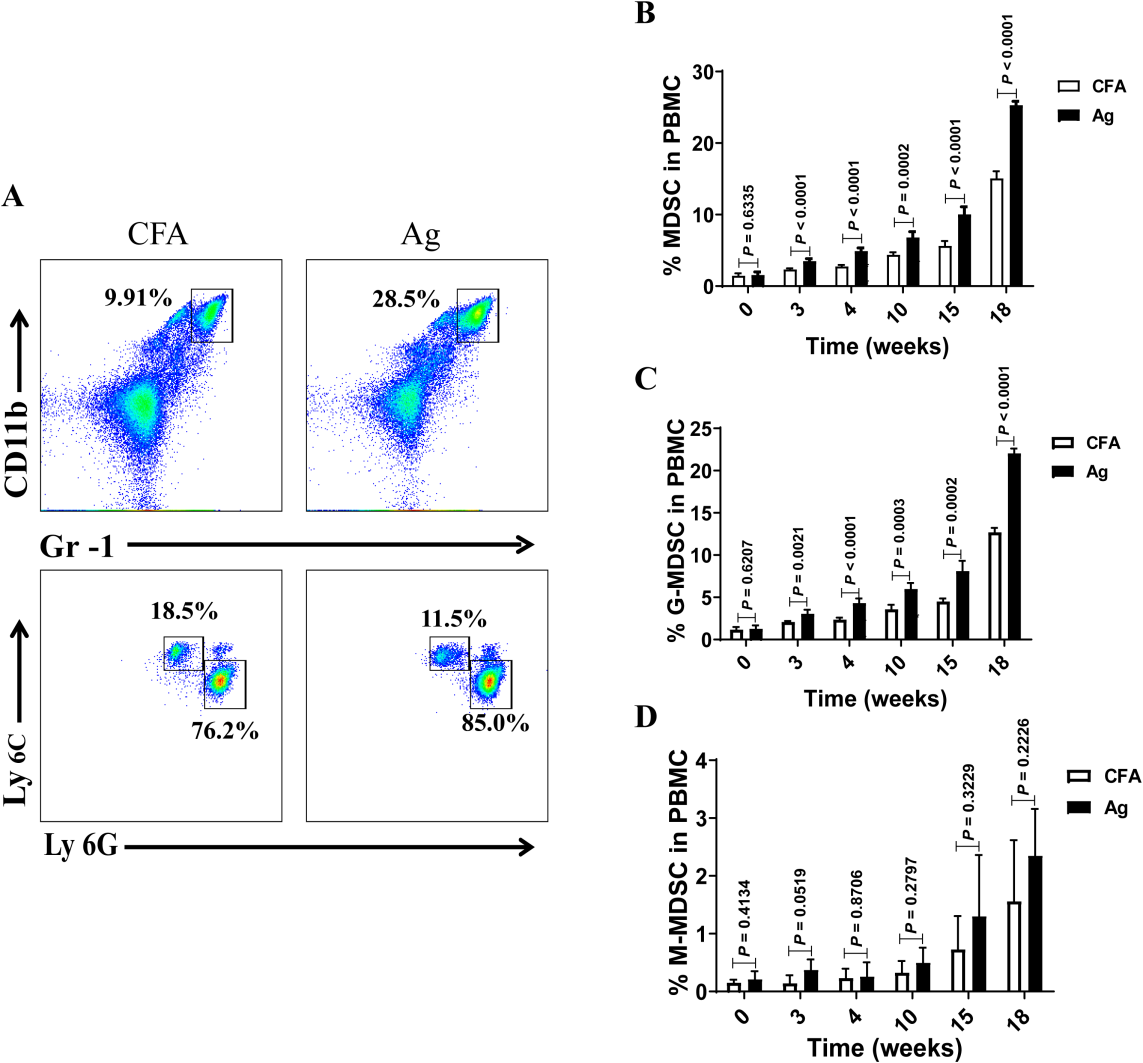
The percentage of myeloid-derived suppressor cells (MDSCs) and their subsets gradually increased in the peripheral blood of model mice with the extension of immunization time. **(A)** Staining profiles of MDSCs (CD11b^+^Gr-1^+^), granulocytic MDSCs (G-MDSCs, CD11b^+^Gr-1^+^ Ly6G^+^) and monocytic MDSCs (M-MDSCs, CD11b^+^Gr-1^+^ Ly6C^+^) from the representative Complete Freund’s Adjuvant (CFA) control group and primary membranous nephropathy (PMN) model (Ag) mice **(B–D)** Percentage of **(B)** MDSCs (Mann-Whitney U test, n = 5 per group; *P* = 0.6335 at 0 W; *P* < 0.0001 at 3 W; *P* < 0.0001 at 4 W; *P* = 0.0002 at 10 W; *P* < 0.0001 at 15 W; *P* < 0.0001 at 18 W), **(C)** G-MDSCs (Mann-Whitney U test, n = 5 per group; *P* = 0.6207 at 0 W; *P* = 0.0021 at 3 W; *P* < 0.0001 at 4 W; *P* = 0.0003 at 10 W; *P* = 0.0002 at 15 W; *P* < 0.0001 at 18 W), and **(D)** M-MDSCs (Mann-Whitney U test, n = 5 per group; *P* = 0.4134 at 0 W; *P* = 0.0519 at 3 W; *P* = 0.8706 at 4 W; *P* = 0.2797 at 10 W; *P* = 0.3229 at 15 W; *P* = 0.2226 at 18 W) in CFA and Ag mice at different immunization time points. The data represent three independent experiments with similar results.

Based on the experimental method of Zhang et al. (11), a murine PMN model was successfully constructed based on urinary ACR, plasma urea levels, and typical pathological changes of PMN (**Supplementary Figure S1**). Our previous studies demonstrated a significant increase in the percentage and number of MDSCs, which positively correlated with disease activity in patients. Blood samples were collected from the mandibles of mice every 3 or every 6 weeks to monitor MDSC levels during the pathological progression of PMN. MDSCs were defined as CD11b^+^Gr-1^+^ cells and further divided into CD11b^+^Gr-1^+^ Ly6C^+^ monocytic MDSCs (M-MDSC) and CD11b^+^Gr-1^+^ Ly6G^+^ granulocytic MDSCs (G-MDSC) subsets (**Figure 1A**). The results showed that the proportions of MDSCs (**Figure 1B**) and G-MDSCs (**Figure 1C**) gradually increased in the peripheral blood of Ag model mice with extension of immunization time, with statistically significant differences compared with those of Complete Freund’s Adjuvant (CFA) mice. However, no significant difference was observed in the proportion of M-MDSCs (**Figure 1D**) between Ag and CFA mice. All mice were sacrificed at 18 weeks of age, and their spleens were collected to prepare single-cell suspensions. Changes in the proportions of MDSCs, G-MDSCs, and M-MDSCs were detected using flow cytometry. The results showed that the proportions of MDSCs and G-MDSCs in the model group increased compared to those in CFA- and gemcitabine-treated mice. Still, the proportion of M-MDSCs was not significantly different (**Supplementary Figure S2**). These findings are consistent with those obtained from peripheral blood analyses.

### 3.2 Enhanced Th17 and Th2 responses correlate with disease progression in the PMN model mice

**Figure 2:**
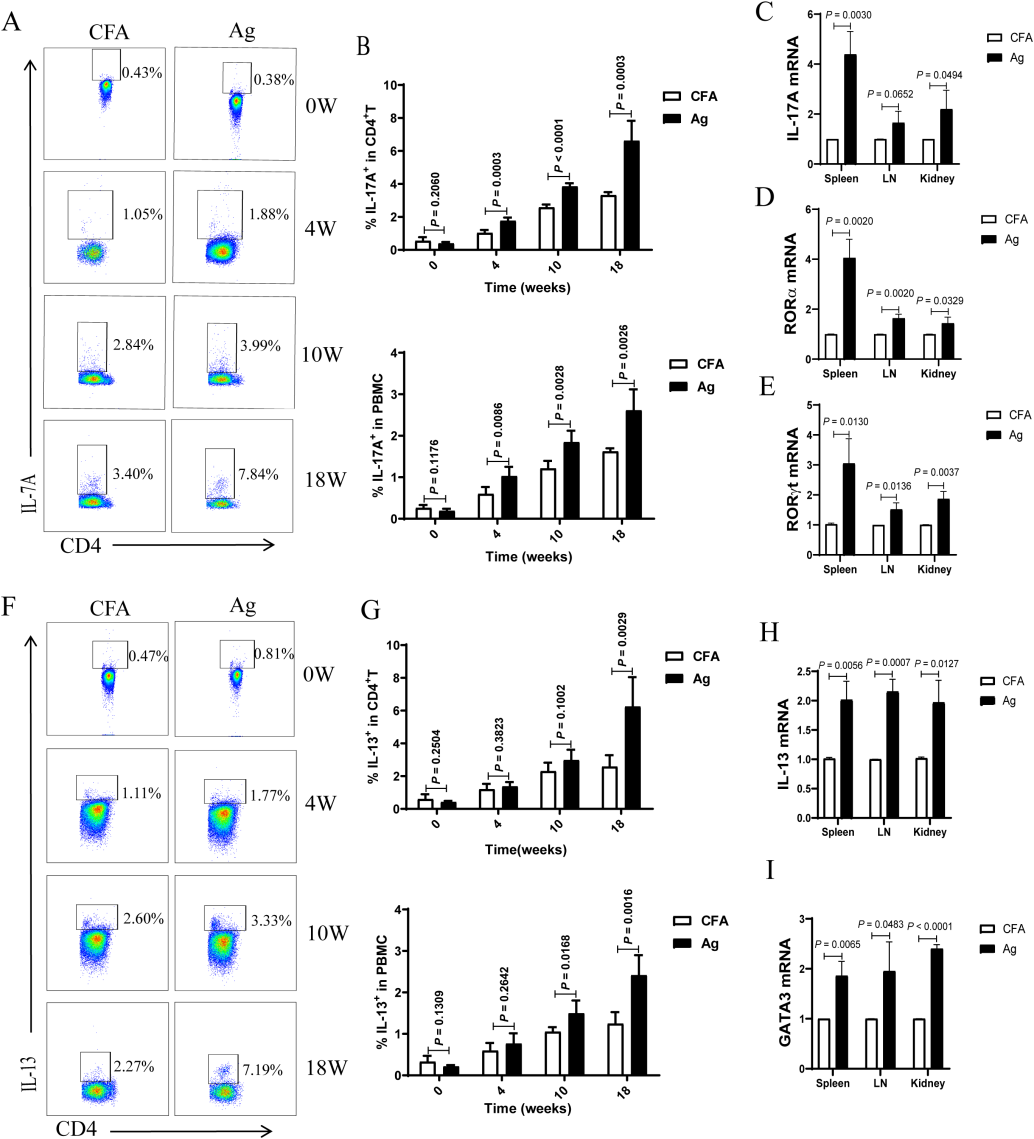
The proportion of T helper 17 (Th17) and T helper 2 (Th2) cells and the mRNA expression of related factors in the peripheral blood of primary membranous nephropathy (PMN) mice increased with the extension of immunization time. **(A, F)** Representative staining profile of interleukin (IL)-17A **(A)** and IL-13 **(F)** in the peripheral blood of Complete Freund’s Adjuvant (CFA) control and primary membranous nephropathy (PMN) model (Ag) mice at different immunization time points **(B)** Percentage of IL-17A in CD4^+^ T cells (upper panel: Mann-Whitney U test, n = 5 per group; *P* = 0.2060 at 0 W; *P* = 0.0003 at 4 W; *P* < 0.0001 at 10 W; *P* = 0.0003 at 18W) and peripheral blood mononuclear cells (PBMCs) (lower panel: Mann-Whitney U test, n = 5 per group; *P* = 0.1176 at 0 W; *P* = 0.0086 at 4 W; *P* = 0.0028 at 10 W; *P* = 0.0026 at 18W) from the peripheral blood of CFA control and Ag model mice at different immunization time points **(C–E)** Statistical graphs depicting **(C)** IL-17A (Mann-Whitney U test, n = 3; *P* = 0.0030 in the spleen; *P* = 0.0652 in the LN; *P* = 0.0494 in the kidney), **(D)** RORα (Mann-Whitney U test, n = 3; *P* = 0.0020 in the spleen; *P* = 0.0020 in the LN; *P* = 0.0329 in the kidney), and **(E)** RORγt (Mann-Whitney U test, n = 3; *P* = 0.0130 in the spleen; *P* = 0.0136 in the LN; *P* = 0.0037 in the kidney) mRNA expression in the spleen, lymph nodes(LN), and kidneys **(G)** Percentage of IL-13 in CD4^+^ T cells (upper panel: Mann-Whitney U test, n = 5 per group; *P* = 0.2504 at 0 W; *P* = 0.3823 at 4 W; *P* = 0.1002 at 10 W; *P* = 0.0029 at 18W) and peripheral blood mononuclear cells (PBMCs) (lower panel: Mann-Whitney U test, n = 5 per group; *P* = 0.1309 at 0 W; *P* = 0.2642 at 4 W; *P* = 0.0168 at 10 W; *P* = 0.0016 at 18W) from the peripheral blood of CFA control and Ag model mice at different immunization time points **(H, I)** Statistical graphs representing **(H)** IL-13 (Mann-Whitney U test, n = 3; *P* = 0.0056 in the spleen; *P* = 0.0007 in the LN; *P* = 0.0127 in the kidney) and **(I)** GATA3 (Mann-Whitney U test, n = 3; *P* = 0.0065 in the spleen; *P* = 0.0483 in the LN; *P* < 0.0001 in the kidney) mRNA expression in the spleen, lymph nodes(LN), and kidneys. The data represent three independent experiments.

Our study showed that Th2 and Th17 immune responses in the peripheral blood of patients with PMN were enhanced and positively correlated with disease activity. Th17 cells are the primary pathogenic T-cell subset in kidney disease, and interleukin (IL)-17 has been shown to damage renal parenchymal cells, leading to renal injury directly. Th1, Th2, and Th17 immune responses play critical roles in autoimmune renal diseases (24–36). Therefore, we examined changes in peripheral blood Th17 and Th2 cells at different time points. The results showed that the percentages of Th17 (CD4^+^ T cells that produce IL-17A) (**Figures 2A, B**) and Th2 (CD4^+^ T cells that produce IL-13) (**Figures 2F, G**) cells gradually increased with the extension of immunization time, surpassing those in the CFA control group after 4 weeks of immunization. The expression levels of IL-17A (**Figures 2C**) and IL-13 (**Figures 2H**) and transcription factors RORα (**Figures 2D**), RORγt (**Figures 2E**), and GATA3 (**Figures 2I**) were significantly elevated in the spleen, lymph nodes, and kidneys of mice in the model group compared to those in CFA mice. These findings indicate that Th17 and Th2 immune responses intensify as membranous nephropathy progresses.

### 3.3 Weakened Th1 and Treg responses correlate with disease progression in the PMN model mice

**Figure 3:**
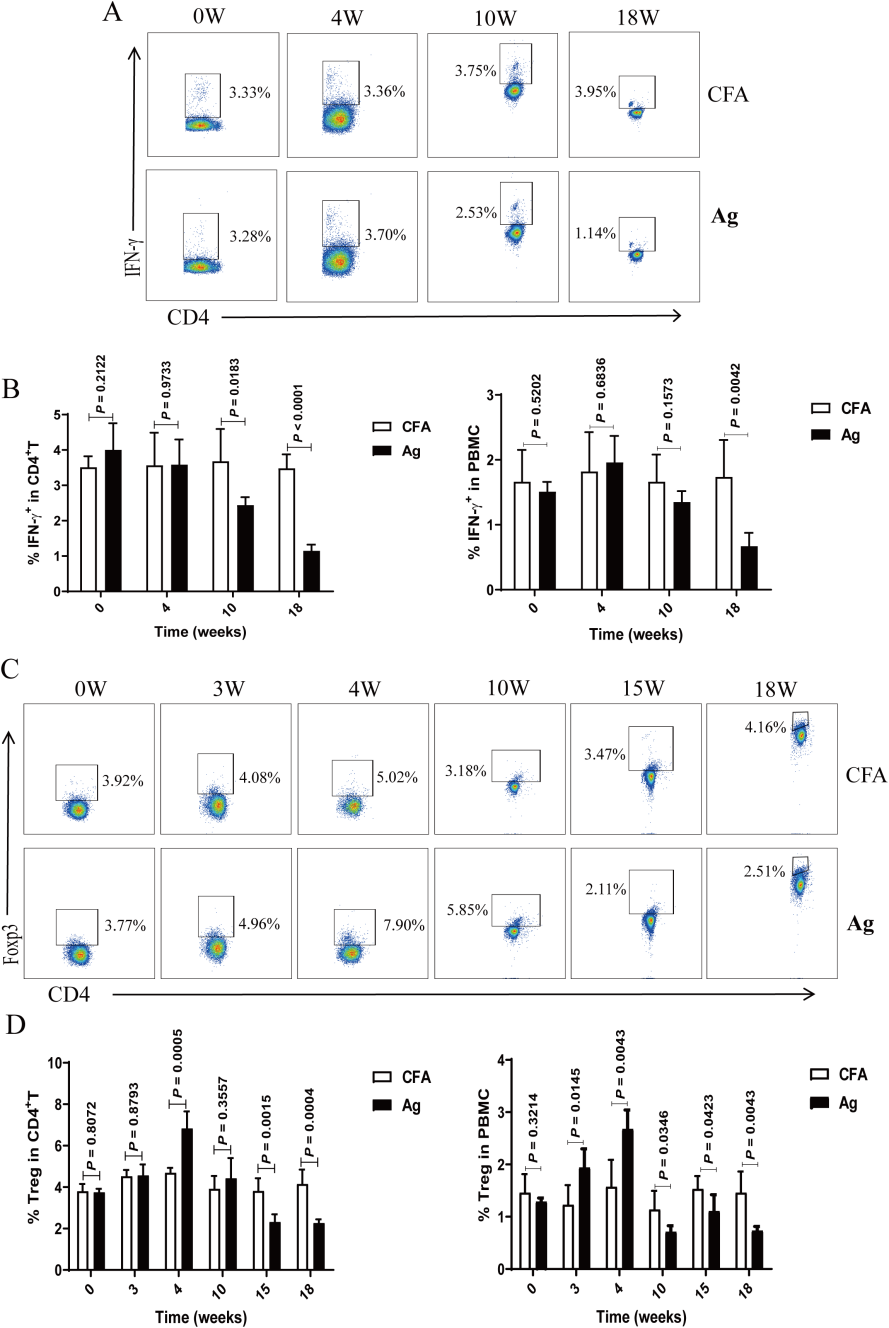
The proportion of T helper 1 (Th1) and regulatory T (Treg) cells in the peripheral blood of primary membranous nephropathy (PMN) mice decreased with the extension of immunization time. **(A, C)** Representative staining profiles of interferon (IFN)-γ **(A)** and Tregs **(C)** in the peripheral blood of Complete Freund’s Adjuvant (CFA) control and primary membranous nephropathy (PMN) model (Ag) mice at different immunization time points **(B, D)** Percentage of **(B)** IFN-γ (Mann-Whitney U test, n = 5 per group; left panel: *P* = 0.2122 at 0 W; *P* = 0.9733 at 4 W; *P* = 0.0183 at 10 W; *P* < 0.0001 at 18W; right panel: *P* = 0.5202 at 0 W; *P* = 0.6836 at 4 W; *P* = 0.1573 at 10 W; *P* = 0.0042 at 18W) and **(D)**Tregs (Mann-Whitney U test, n = 5 per group; left panel: *P* = 0.8072 at 0 W; *P* = 0.8793 at 3 W; *P* = 0.0005 at 4 W; *P* = 0.3557 at 10 W; *P* = 0.0015 at 15 W; *P* = 0.0004 at 18 W; right panel: *P* = 0.3214 at 0 W; *P* = 0.0145 at 3 W; *P* = 0.0043 at 4 W; *P* = 0.0346 at 10 W; *P* = 0.0423 at 15 W; *P* = 0.0043 at 18 W) in CD4^+^ T cells (left panel) and peripheral blood mononuclear cells (PBMCs) (right panel) from the peripheral blood of CFA control and Ag model mice at different immunization time points. The data represent three independent experiments.

Similarly, we analyzed the temporal changes in peripheral blood Th1 (CD4^+^ T cells that produce IFN-γ) and Treg cells levels. Compared to that in CFA mice, the percentage of Th1 cells progressively declined with the extension of immunization time (**Figures 3A, B**). The percentage of Tregs exhibited a transient increase at 4 weeks but subsequently declined, becoming significantly lower than that in the CFA control mice at 15 and 18 weeks (**Figures 3C, D**).

### 3.4 Gemcitabine effectively depletes MDSCs in the peripheral blood of model mice

**Figure 4:**
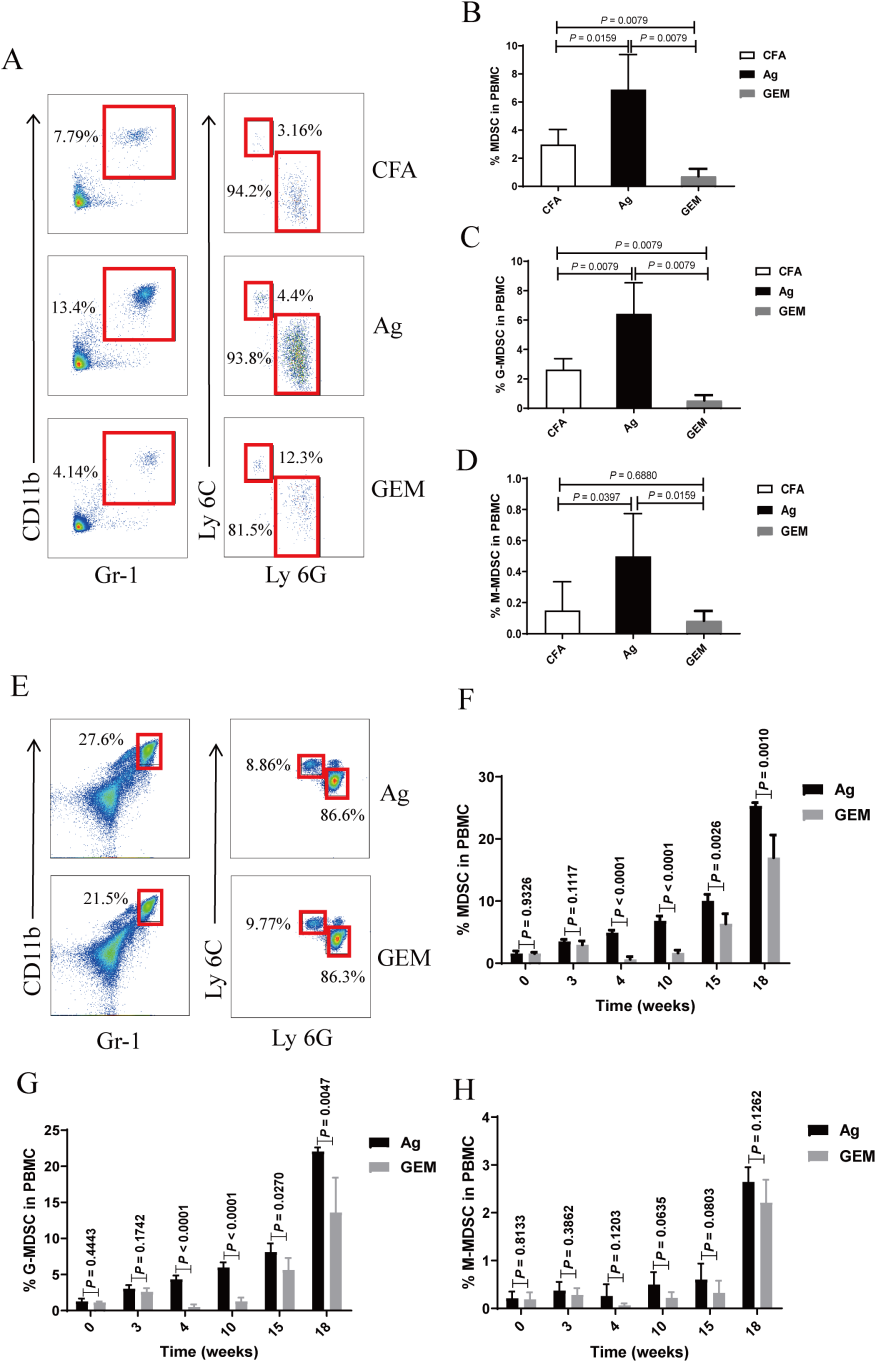
The percentage of myeloid-derived suppressor cells (MDSCs) and their subsets was significantly reduced in the peripheral blood of model mice following gemcitabine treatment. **(A–D)** Staining profiles **(A)** and percentages of **(B)** MDSCs (One-way ANOVA, Tukey’s *post-hoc* test, n = 5 per group; CFA vs. Ag, *P* = 0.0159; Ag vs. GEM, *P* = 0.0079; CFA vs. GEM, *P* = 0.0079), **(C)** granulocytic (G)-MDSCs (One-way ANOVA, Tukey’s *post-hoc* test, n = 5 per group; CFA vs. Ag, *P* = 0.0079; Ag vs. GEM, *P* = 0.0079; CFA vs. GEM, *P* = 0.0079), and **(D)** monocytic (M)-MDSCs (One-way ANOVA, Tukey’s *post-hoc* test, n = 5 per group; CFA vs. Ag, *P* = 0.0397; Ag vs. GEM, *P* = 0.0159; CFA vs. GEM, *P* = 0.6880) in the peripheral blood of representative Complete Freund’s Adjuvant (CFA), primary membranous nephropathy (PMN) model (Ag) mice, and Gemcitabine-treated (GEM) mice on day 2 after intraperitoneal injection of gemcitabine **(E–H)** Staining profiles **(E)** and percentages of **(F)** MDSCs (Mann-Whitney U test, n = 5 per group; *P* = 0.9326 at 0 W; *P* = 0.1117 at 3 W; *P* < 0.0001 at 4 W; *P* < 0.0001 at 10 W; *P* = 0.0026 at 15 W; *P* = 0.0010 at 18 W), **(G)** G-MDSCs (Mann-Whitney U test, n = 5 per group; *P* = 0.4443 at 0 W; *P* = 0.1742 at 3 W; *P* < 0.0001 at 4 W; *P* < 0.0001 at 10 W; *P* = 0.0270 at 15 W; *P* = 0.0047 at 18 W), and **(H)** M-MDSCs (Mann-Whitney U test, n = 5 per group; *P* = 0.8133 at 0 W; *P* = 0.3862 at 3 W; *P* = 0.1203 at 4 W; *P* = 0.0635 at 10 W; *P* = 0.0803 at 15 W; *P* = 0.1262 at 18 W) in the peripheral blood of representative Ag and GEM mice at different time points after immunization. The data represent three independent experiments.

Gemcitabine specifically reduces the number of MDSCs in tumor-bearing mice without significantly decreasing the number of T cells, natural killer (NK) cells, macrophages, or B cells (27–29). To investigate the relationship between MDSCs and PMN in a mouse model, we administered gemcitabine. Treatment with 100 mg/kg gemcitabine effectively reduced the percentages of MDSCs, G-MDSCs, and M-MDSCs in the peripheral circulation compared to those in the CFA and Ag groups (**Figures 4A-D**). The proportions of MDSCs, G-MDSCs, and M-MDSCs in the gemcitabine-treated group were lower than those in the Ag model group 4 weeks after immunization (**Figures 4E-H**).

### 3.5 MDSC depletion with gemcitabine alleviates renal tissue damage in the PMN model mice

**Figure 5:**
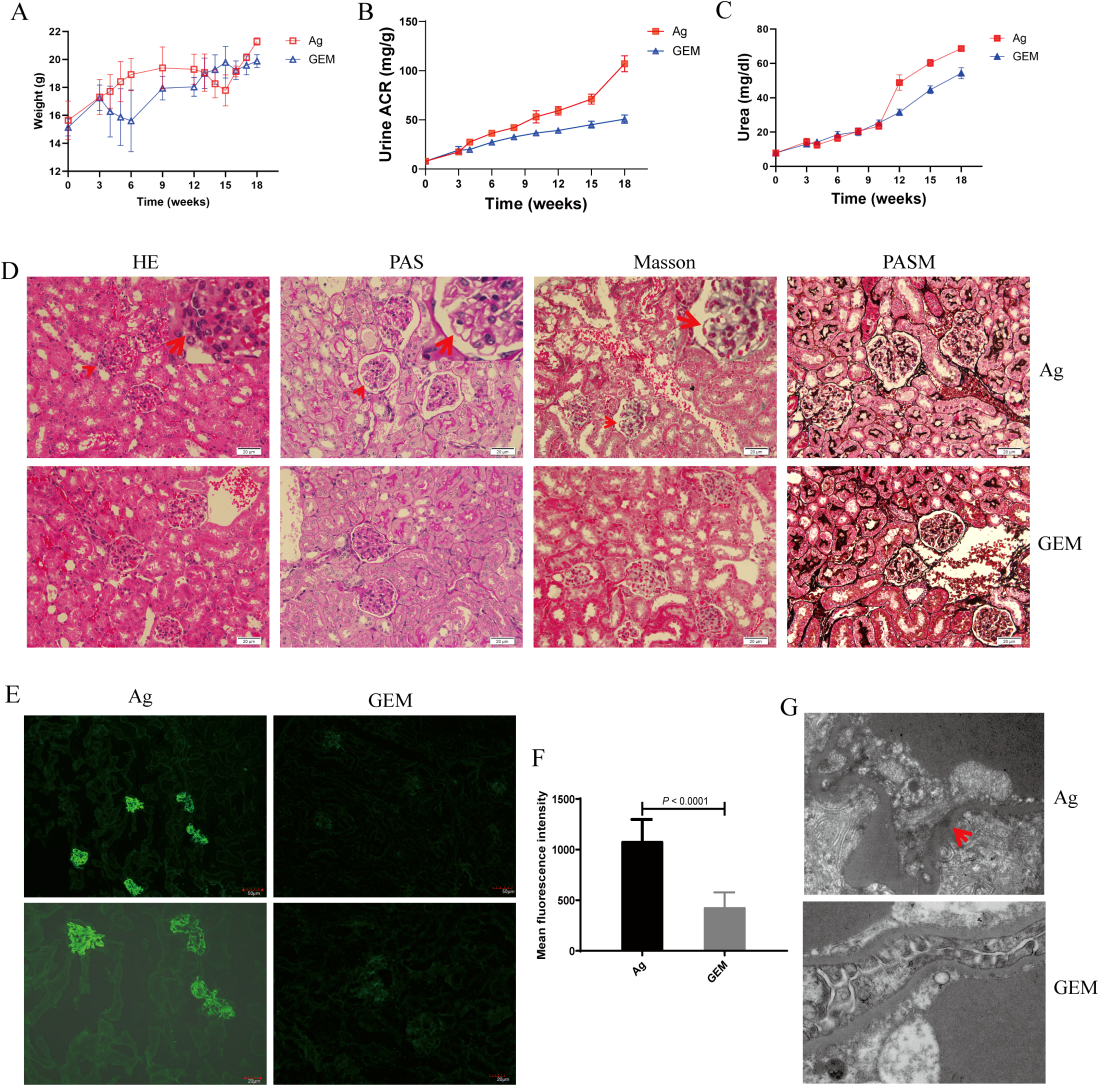
Urine albumin-to-creatinine ratio (ACR) and plasma urea levels decreased, and renal tissue pathology improved following myeloid-derived suppressor cells (MDSCs) depletion. **(A)** Body weight changes in primary membranous nephropathy (PMN) model (Ag) mice and Gemcitabine-treated (GEM) model mice **(B, C)** Urine albumin-to-creatinine ratio (ACR) **(B)** and plasma urea levels **(C)** were lower in the GEM group than in the Ag model mice (Ag: red lines; GEM: blue lines); **(D)** Focal inflammatory cell infiltration outside the arteriolar capsule (arrow) in the H&E-stained section, mesangial stiff in the glomerulus (arrow) identified by PAS staining, immune complex deposition in the basal membrane by Masson are shown. Representative images are shown in Ag mice. No stiff glomerular capillary loops or subepithelial electron-dense deposits were observed in GEM mice. Light microscopic images of renal tissue (upper panel: Ag model group; lower panel: GEM group). Original magnification: 400× **(E)** Representative immunofluorescence (IF) staining images of immunoglobulin G (IgG) in kidney tissue sections from Ag and GEM mice (left panel: Ag model group; right panel: GEM group). Scale bars: 50 μm (top) and 20 μm (bottom) **(F)** Mean fluorescence intensity (per μm^2^) of IgG. Sixteen randomly selected areas of kidney tissues from Ag and GEM mice were analyzed using a Mann-Whitney U test (*P* < 0.0001) **(G)** Transmission electron microscopy (TEM) images shows subepithelial electron-dense deposits (arrow), and podocyte foot process fusion in Ag model mice. The absence of electron-dense deposits and stiff glomerular capillary loops was observed in GEM mice (upper panel: Ag model group; lower panel: GEM group). Original magnification: 4000×.

The body weights of Ag- and gemcitabine-treated mice were monitored weekly. The results showed that the weight of mice in the model group began to decline at 12 weeks. After 15 weeks, body weight recovered and gradually increased. In contrast, mice in the gemcitabine -treated (GEM) group showed a significant decrease in body weight after gemcitabine injection. Body weight gradually recovered and continued to increase after 6 weeks, albeit at a slower rate than that in the model group (**Figure 5A**). Urine albumin-to-creatinine ratio (ACR) and plasma urea levels in the GEM-treated group slowly increased but remained lower than those in the Ag model group (**Figures 5B, C**). No significant immune complex deposition was observed in the glomerular basement membrane (GBM) of the renal tissue in the gemcitabine group (**Figure 5D**). Weak immunofluorescence staining was detected in the gemcitabine group, with a significantly lower fluorescence intensity than that in the model group (**Figures 5E, F**). TEM revealed that the absence of electron-dense deposits and stiff glomerular capillary loops was observed in GEM mice (**Figure 5G**). The specific lesion score of Ag model and GEM mice was shown by **Supplementary Figure S6**. These findings indicated that urinary ACR and plasma urea levels were reduced, and renal tissue pathological injury was significantly alleviated after MDSCs depletion.

### 3.6 MDSC depletion with gemcitabine attenuated Th17 and Th2 immune responses in the PMN mice

**Figure 6:**
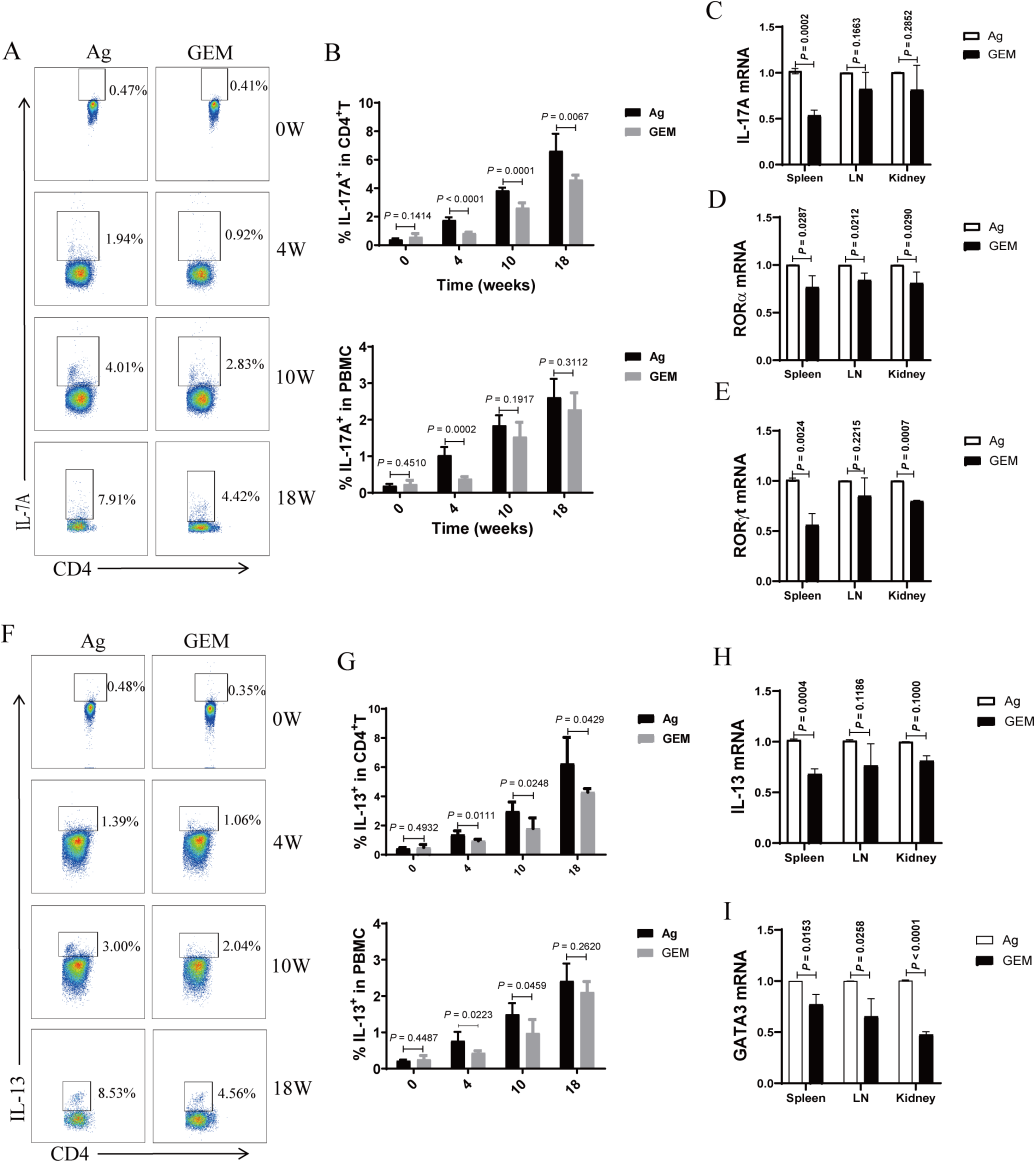
Myeloid-derived suppressor cells (MDSCs) depletion with gemcitabine reduced the proportion of T helper 17 (Th17) and T helper 2 (Th2) cells and the mRNA expression of interleukin (IL)-17A and IL-13 cytokines and their associated transcription factors in primary membranous nephropathy (PMN) model mice. **(A, F)** Representative staining profiles of IL-17A **(A)** and IL-13 **(F)** at different time points **(B)** Statistical charts: the upper panel shows the percentage of IL-17A in CD4^+^ T cells (Mann-Whitney U test, n = 5 per group; *P* = 0.1414 at 0 W; *P* < 0.0001 at 4 W; *P* = 0.0001 at 10 W; *P* = 0.0067 at 18 W); the lower panel shows the percentage of IL-17A in peripheral blood mononuclear cells (PBMCs) (Mann-Whitney U test, n = 5 per group; *P* = 0.4510 at 0 W; *P* = 0.0002 at 4 W; *P* = 0.1917 at 10 W; *P* = 0.3112 at 18W) **(C–E)** Statistical graphs depicting **(C)** IL-17A (Mann-Whitney U test, n = 3; *P* = 0.0002 in the spleen; *P* = 0.1663 in the LN; *P* = 0.2852 in the kidney), **(D)** RORα (Mann-Whitney U test, n = 3; *P* = 0.0287 in the spleen; *P* = 0.0212 in the LN; *P* = 0.0290 in the kidney), and **(E)** RORγt (Mann-Whitney U test, n = 3; *P* = 0.0024 in the spleen; *P* = 0.2215 in the LN; *P* = 0.0007 in the kidney) mRNA expression levels in the spleen, lymph nodes (LN), and kidneys **(G)** Statistical charts: the upper panel shows the percentage of IL-13 in CD4^+^ T cells (Mann-Whitney U test, n = 5 per group; *P* = 0.4932 at 0 W; *P* = 0.0111 at 4 W; *P* = 0.0248 at 10 W; *P* = 0.0429 at 18 W); the lower panel shows the percentage of IL-13 in PBMCs (Mann-Whitney U test, n = 5 per group; *P* = 0.4487 at 0 W; *P* = 0.0223 at 4 W; *P* = 0.0459 at 10 W; *P* = 0.2620 at 18 W) **(H, I)** Statistical graphs illustrating **(H)** IL-13 (Mann-Whitney U test, n = 3; *P* = 0.0004 in the spleen; *P* = 0.1186 in the LN; *P* = 0.1000 in the kidney)and **(I)** GATA3 (Mann-Whitney U test, n = 3; *P* = 0.0153 in the spleen; *P* = 0.0258 in the LN; *P* < 0.0001 in the kidney) mRNA expression levels in the spleen, lymph nodes (LN), and kidneys. The data represent three independent experiments.

We analyzed the percentages of Th17 and Th2 cells in circulation, as well as the expression levels of IL-17A and IL-13 cytokines and their related transcription factors—RORγt, RORα, and GATA3 mRNA—in the spleen, lymph nodes, and kidneys of PMN model mice after MDSC depletion with gemcitabine. The percentages of Th17 (**Figures 6A, B**) and Th2 (**Figures 6F, G**) cells increased with increasing molding time. However, compared to that in the Ag model mice, MDSC depletion at 4, 10, and 18 weeks led to a reduction in the percentage of Th17 and Th2 cells. In addition, the expression levels of IL-17A (**Figure 6C**) and IL-13 (**Figure 6H**) cytokines, along with their associated transcription factors RORα (**Figure 6D**), RORγt (**Figure 6E**), and GATA3 mRNA (**Figure 6I**)), were significantly reduced in the spleen, lymph nodes, and kidneys of gemcitabine-treated mice after MDSC depletion at 18 weeks.

### 3.7 MDSC depletion with gemcitabine enhanced the Th1 immune response and Treg ratio in PMN mice

**Figure 7:**
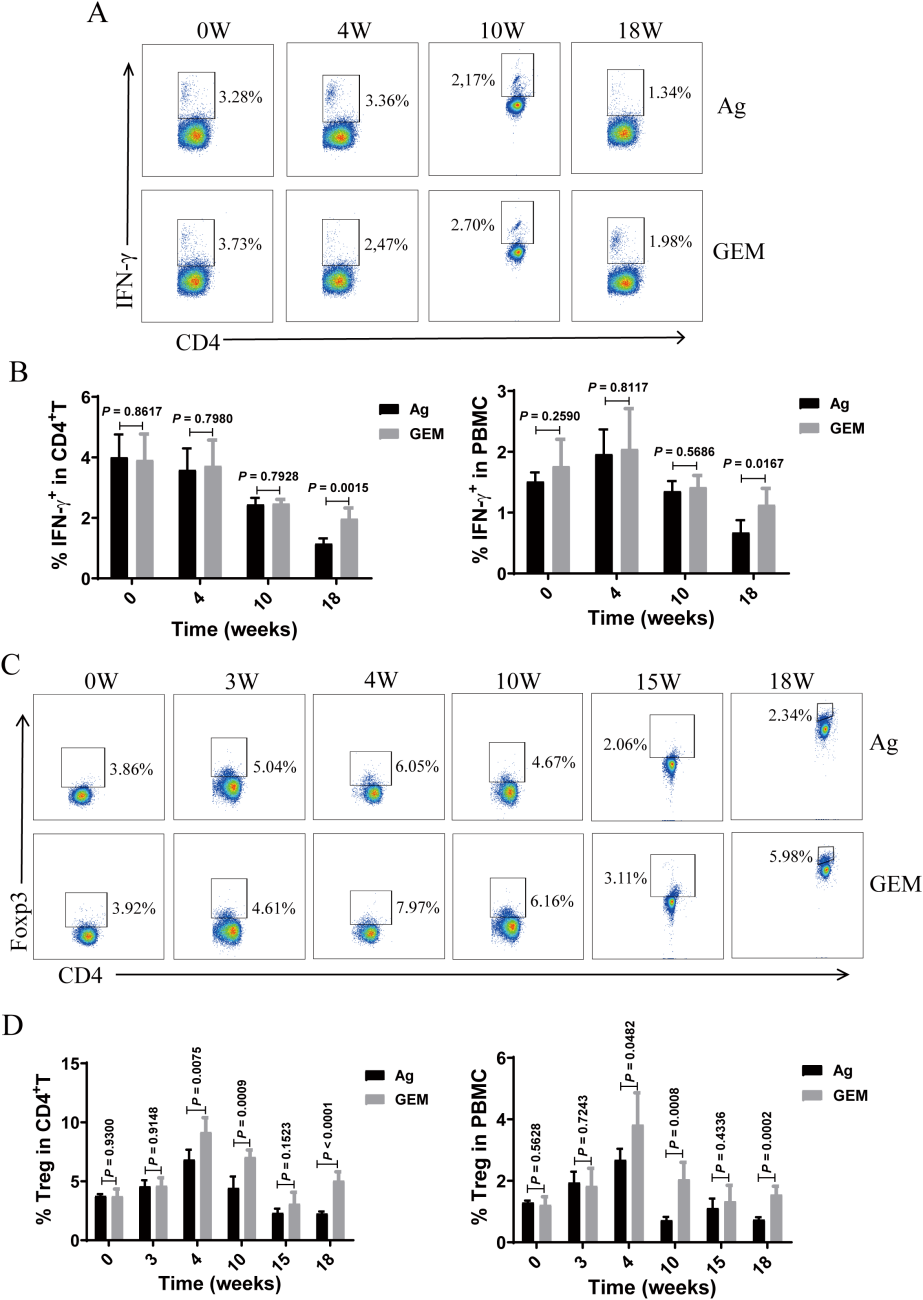
Myeloid-derived suppressor cells (MDSCs) depletion with gemcitabine increased the proportion of Th1 and Treg cells in the primary membranous nephropathy (PMN) model mice. **(A, C)** Representative staining profiles of interferon (IFN)-γ **(A)** and Foxp3 **(C)** in the peripheral blood of primary membranous nephropathy (PMN) model (Ag) mice and Gemcitabine-treated (GEM) model mice at various time points post-immunization **(B)** Statistical charts: left panel shows the percentage of IFN-γ in CD4^+^ T cells (Mann-Whitney U test, n = 5 per group; *P* = 0.8617 at 0 W; *P* = 0.7980 at 4 W; *P* = 0.7928 at 10 W; *P* = 0.0015 at 18 W); right panel shows the percentage of IFN-γ in peripheral blood mononuclear cells (PBMCs) (Mann-Whitney U test, n = 5 per group; *P* = 0.2590 at 0 W; *P* = 0.8117 at 4 W; *P* = 0.5686 at 10 W; *P* = 0.0167 at 18 W) **(D)** Statistical charts: left panel shows the percentage of Tregs in CD4^+^ T cells (Mann-Whitney U test, n = 5 per group; *P* = 0.9300 at 0 W; *P* = 0.9148 at 3 W; *P* = 0.0075 at 4 W; *P* = 0.0009 at 10 W; *P* = 0.1523 at 15 W; *P* < 0.0001 at 18 W); right panel shows the percentage of regulatory T cells (Tregs) in PBMCs (Mann-Whitney U test, n = 5 per group; *P* = 0.5628 at 0 W; *P* = 0.7243 at 3 W; *P* = 0.0482 at 4 W; *P* = 0.0008 at 10 W; *P* = 0.4336 at 15 W; *P* = 0.0002 at 18 W). The data represent three independent experiments.

We determined the percentages of Th1 and Treg cells in the circulation of the PMN model mice before and after MDSC depletion with gemcitabine. The proportion of Th1 cells (**Figures 7A, B**) decreased with increasing molding time. However, compared to that in the Ag model mice, MDSC depletion resulted in an increase in the percentage of Th1 cells in the gemcitabine-treated group at 18 weeks (**Figure 7B**). Experimental animal studies have confirmed that changes in Treg cell numbers and function can influence the severity of nephritis (30–33). Therefore, we monitored Treg cell percentages in the peripheral blood at various time points post-immunization. In gemcitabine-treated mice, the percentage of Treg cells (**Figures 7C, D**) was significantly elevated at 4 weeks but gradually declined thereafter, reaching lower levels at 15 weeks. However, these levels remained higher than those observed in the Ag model mice.

## 4 Discussion

Our previous research showed that MDSCs promote PMN disease progression by enhancing Th17 response in human PMN diseases (21), we want to further verify their relationship between MDSCs and Th17 cells in murine models in the occurrence and development of PMN diseases. Meanwhile, the relationship between T cells and PMN diseases was further verified after MDSCs depletion with gemcitabine. This study investigated, for the first time, the relationship between MDSCs and T cells in a murine model. We observed an increase in the proportion of MDSCs and their subclasses, as well as Th17 and Th2 cells, along with elevated mRNA expression of related factors. Conversely, the proportions of Th1 and Treg cells in the peripheral blood decreased as the immunization time progressed in the PMN model mice. After MDSC depletion with gemcitabine in a murine PMN model, renal tissue damage was alleviated, Th17 and Th2 immune responses, and the expression of associated transcription factors decreased. In contrast, the Th1 immune response and Treg ratio were enhanced.

Research on the pathogenesis and development of novel targeted therapies for PMN has been hampered by the lack of reliable animal models (34). Among the available models, rat Heymann nephritis is considered the most representative model for studying PMN pathogenesis, owing to its close resemblance to human MN. However, this model has several limitations (35). Currently, three primary murine models have been developed to simulate PMN pathology using established technologies accurately (36, 37). Considering the advantages and disadvantages of these murine models and their laboratory feasibility, we selected a murine model of PMN induced by type IV collagen fragments for this experimental study.

MDSCs were initially identified in patients with cancer and tumor-bearing mice and are now recognized for their roles in cancer, inflammation, infection, and autoimmune diseases (38, 39). Several studies have demonstrated that MDSCs contribute to the progression of glomerulonephritis (40, 41), regulating immune responses by suppressing T cell proliferation, interfering with T cell trafficking and viability, and inducing the differentiation of Treg cells (42). Shi et al. (43) reported that in the circulation of patients with idiopathic MN (IMN), the ratio of CD4^+^/CD8^+^ T cells to PD-L1^+^ M-MDSCs was elevated. In contrast, the proportions of Tregs and G-MDSCs were reduced compared to those of healthy controls. In patients with PLA2R-positive IMN, the ratio of CD4^+^/CD8^+^ T cells was higher, whereas the frequencies of PD-1^+^CD4^+^ T cells, CTLA-4^+^CD4^+^ T cells, PD-1^+^ Tregs, and CTLA-4^+^ Tregs were lower in PBMCs compared with those in patients with PLA2R-negative IMN. These findings suggest that MDSCs and T cell subsets are involved in the pathogenesis of MN. Our previous study further confirmed that the MDSC ratio is significantly elevated in patients with PMN and is positively correlated with disease activity (21). Similarly, in this study, we observed an increase in the proportions of MDSCs and G-MDSCs in the PMN model group. In contrast, the proportion of M-MDSCs remained unchanged after prolonged immunization.

MDSCs promote disease progression by enhancing Th17 differentiation in various autoimmune diseases, including SLE (14), RA (15, 16), and EAE (44). Studies have confirmed that Th17 cells play a key role in IMN pathogenesis. These cells differentiate in response to TGF-β, IL-6, and IL-17 to produce IL-17A and IL-21, which facilitate the recruitment of other inflammatory cells and directly contribute to renal tissue damage (45). T cells and their subsets, including Th2, Th17, follicular helper T (Tfh), and Treg cells, contribute to immune imbalance in the IMN and promote the incidence and progression of autoimmune responses. Similarly, Th cells contribute to immune dysregulation in the IMN and the production of IMN-specific antibodies. In IMN, Th cell subpopulations are predominantly composed of Th2, Th17, and Tfh cells, whereas Treg and Th1 cells are impaired (46–48). Compared to healthy individuals, patients with MN exhibit elevated serum levels of Th17 and Th2 cytokines, including IL-17A, IL-6, and IL-4, alongside deficiencies in Th1 and Treg cells cytokines, such as IFN-γ and IL-10. Moreover, patients with PMN- and Th17-mediated inflammation experience higher rates of venous thromboembolic events, more frequent relapses, and poorer prognoses (49). An altered Th17/Treg ratio has been proposed as a potential mechanism underlying IMN pathogenesis (50). Our current study showed that the proportion of Th17 cells in the peripheral blood of PMN mice, along with the mRNA expression of IL-17A, RORγt, and RORα in the spleen, lymph nodes, and kidneys, increased with prolonged immunization. MDSC depletion with gemcitabine reduced the proportion of Th17 cells and the mRNA expression of IL-17A and its associated transcription factors in a murine PMN model. These findings suggest that MDSC and Th17 responses play crucial roles in PMN progression, providing new insights into its pathogenesis and potential clinical treatment strategies.

Our study confirmed a predominant Th2 immune response in a murine PMN model induced by rh-α3NC1 immunization in DBA/1 mice, consistent with the findings of a previous study by Zhang et al. (11). The CD4^+^/CD8^+^ ratio may serve as a predictive marker for response to immunosuppressive therapy by impairing inhibitory T cell function and contributing to proteinuria (51). Kuroki et al. also found that the Th1/Th2 ratio is closely associated with PMN (52). Our results further confirmed that the proportion of Th2 cells and mRNA expression of IL-13 and its associated transcription factors were significantly increased in the PMN mouse model. In contrast, the proportions of Th1 and Treg cells in the peripheral blood of PMN mice decreased as immunization time progressed.

Gemcitabine is a clinically approved antitumor agent (35). A previous study showed that gemcitabine effectively reduced the number of MDSCs in tumor-bearing and EAE mice without significantly decreasing the number of T cells, NK cells, macrophages, or B cells (17, 27–29). The study demonstrated that disease progression in the murine PMN model group worsened as the immunization time increased, accompanied by a gradual increase in the proportion of MDSCs in the peripheral blood. When the mice were sacrificed at 18 weeks, the number of MDSCs was significantly elevated in both the peripheral blood and spleen. The Th17 and Th2 immune responses were enhanced, whereas the Th1 immune response and Treg ratio were downregulated. After MDSC depletion with gemcitabine, the proportion of MDSCs decreased, and renal pathological changes were alleviated. The Th17 and Th2 immune responses were weakened, whereas the Th1 immune response and Treg ratio were enhanced. In the spleen and lymph nodes of gemcitabine-treated mice, the proportion of Th2 and Th17 cells decreased (**Supplementary Figure S3**), whereas that of Th1 and Treg cells increased (**Supplementary Figure S4**). These findings suggest that MDSCs play a critical role in murine PMN by regulating T-cell differentiation. MDSCs may contribute to disease progression by enhancing Th17 and Th2 immune responses while suppressing the Th1 immune response. These results are consistent with those of previous studies in human patients with PMN (21). Our findings indicated that gemcitabine may serve as a potential treatment for PMN, offering a new avenue for research.

Studies on B and T cell responses to rituximab therapy in PMN have demonstrated that monitoring Treg changes may help predict and assess early rituximab responses before the PLA2R-Ab levels decline (51). The percentage of Tregs exhibited a transient increase at 4 weeks but subsequently declined, becoming significantly lower than that in the CFA control mice at 15 and 18 weeks (**Figures 3C, D**). We speculate that the model mice were first injected with gemcitabine on the 25th day, which inhibited the function of MDSCs and caused a temporary increase in the proportion of Tregs. And adaptive remodeling of the intracellular microenvironment of the model mice, which released cytokines and promoted the differentiation and aggregation of Tregs. This might be a negative feedback regulation of the immune activation in the mouse body during the initial stage of gemcitabine treatment. Studies have shown that during the treatment of pancreatic cancer with gemcitabine, the ratio of effector T cells to regulatory T cells increased after just one treatment (53). Gemcitabine can inhibit MDSCs (especially G-MDSCs) or directly kill tumor cells, thereby releasing tumor antigens and activating effector T cells. At this time, the compensatory increase of Tregs can limit excessive immune responses and avoid autoimmune damage or inflammatory storms (28, 54). Other studies have shown that gemcitabine may promote the differentiation and recruitment of Tregs by influencing the cytokines (such as GM-CSF, TGF-β) secreted by tumor cells (55). Additionally, under stress conditions, tumor cells may release exosomes or metabolites (such as adenosine), directly inducing the proliferation of Tregs (56).

Our results showed that MDSC depletion with gemcitabine treatment led to an increased proportion of Tregs in mice, suggesting that gemcitabine may alleviate PMN by enhancing Treg numbers and immunosuppressive functions following MDSC depletion. Some results suggested Th17/Treg imbalance is important for PMN pathogenesis in human diseases and animal models (50, 57–59). Our research shows that the Th17/Treg imbalance in murine PMN and after the removal of MDSCs. These results suggest that Tregs may play a significant role in the pathological process of PMN diseases through a certain network regulation with MDSCs and Th17. However, we did not evaluate the functional capacity of the increased Treg population observed after MDSC depletion. In the subsequent experiments, we will focus on supplementing the research on the Treg function experiment, including tissue localization in the kidney tissue, assessment of its inhibitory ability including co-culture experiment and cytokine detection (IL-10 and TGF-β) *in vitro*, and functional verification including adoptive transfer experiment and Tregs depletion experiment with antibody *in vivo.*

## Conclusion

Based on previous studies on MDSCs and T cells in patients with PMN, we investigated the role of MDSCs, their subclasses, and T cell subsets in a murine PMN model. We analyzed the pathological changes in the kidney and alterations in T cells and their subclasses following MDSC depletion. Our findings suggest that MDSCs may contribute to disease progression by influencing T cell differentiation and the immune response, offering novel insights into the etiology, pathogenesis, clinical diagnosis, and treatment of MN. In the future, we will further explore the mechanism of MDSCs, T cells and gemcitabine in PMN diseases and translate them into clinical diagnosis and treatment.

## Data Availability

The original contributions presented in the study are included in the article/**Supplementary Material**. Further inquiries can be directed to the corresponding author.
