# Supplementary material for: Depletion of myeloid-derived suppressor cells alleviates kidney damage in murine membranous nephropathy
Source: Front Immunol. 2025 Aug 27;16:1623613. doi: 10.3389/fimmu.2025.1623613 (PMC12420248; doi:10.3389/fimmu.2025.1623613)
Supplement: Supplementary file 1 [file Supplementaryfile1.pdf]

Supplementary Material

Supplementary Figures

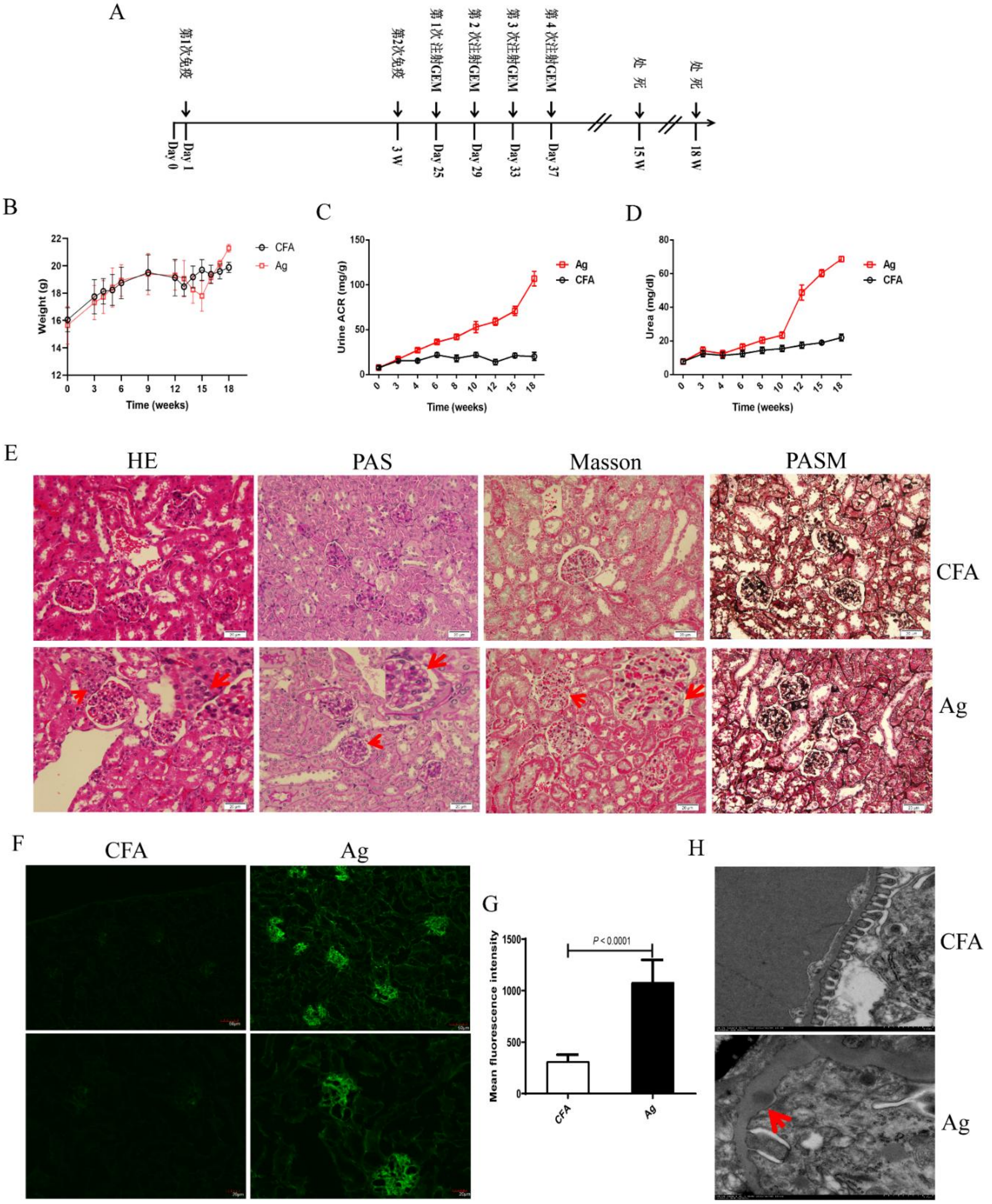

Supplementary Figure 1. Mice immunized with rh-α3NC1 had typical pathological changes of primary membranous nephropathy (PMN).

(A) Time course of rh-α3NC1 immunization and gemcitabine injection in DBA/1 mice **(B)** Body weight change in the Complete Freund's Adjuvant (CFA) and primary membranous nephropathy (PMN) model (Ag) mice **(C and D)** Compared to those in the CFA control group, urine albumin-to-creatinine ratio (ACR) (C) and plasma urea levels (D) were markedly increased in PMN Ag model mice (CFA: black lines; Ag: red lines). **(E)** Focal inflammatory cell infiltration outside the arteriolar capsule (arrow) in the H&E-stained section, mesangial stiff in the glomerulus (arrow) identified by PAS staining, immune complex deposition in the basal membrane by Masson are shown in murine Ag model. Representative images are shown. Light microscopic changes in renal tissue (the upper and lower layers are the control CFA and model Ag groups, respectively)

Original magnification: 400× **(F)** Representative immunofluorescence (IF) staining images of immunoglobulin G (IgG) in kidney tissue sections from the CFA and Ag mice (left panel: control CFA group; right panel: Ag model group)

Scale bars represent 50 μm (top) and 20 μm (bottom), respectively. **(G)** Mean fluorescence density (per μm^2) of IgG

The randomly selected sixteen areas of the kidney tissues from CFA and Ag mice were analyzed using Mann-Whitney U test ($P < 0.0001$) **(H)** Electron microscopy image showing subepithelial electron-dense deposition (red arrow) in the subepithelium, and fusion of podocyte foot processes in Ag model mice. Representative images are shown (upper panel: control CFA group; lower panel: Ag model group)

Original magnification: 4000×

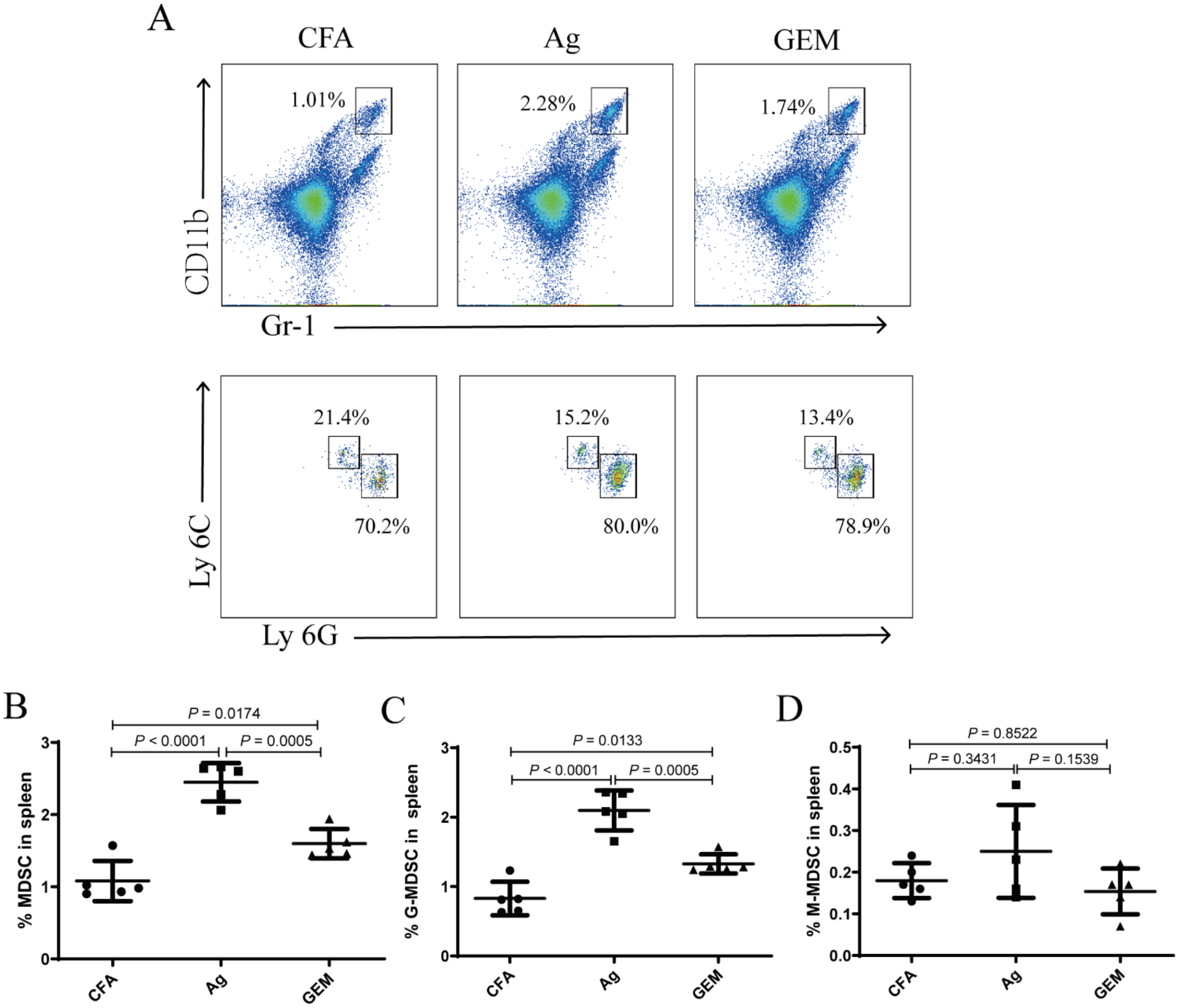

Supplementary Figure 2. The proportion of myeloid-derived suppressor cells (MDSCs) and their subsets in murine spleen from Complete Freund's Adjuvant (CFA), primary membranous nephropathy (PMN) model (Ag) mice, and gemcitabine groups at 18 weeks of immunization.

(A) Staining profiles of MDSCs ($CD11b^{+}Gr\text{-}1^{+}$), granulocytic (G)-MDSCs ($CD11b^{+}Gr\text{-}1^{+}$ Ly6G+), and monocytic (M)-MDSCs ($CD11b^{+}Gr\text{-}1^{+}$ Ly6C+) in the spleen of representative CFA-, Ag-, and gemcitabine-treated (GEM) mice **(B–D)** Percentages of (B)MDSCs (One-way ANOVA, Tukey's post-hoc test, n = 5 per group; CFA vs. Ag , $P < 0.0001$; Ag vs. GEM, $P = 0.0005$; CFA vs. GEM, $P = 0.0174$), (C) G-MDSCs (One-way ANOVA, Tukey's post-hoc test, n = 5 per group; CFA vs. Ag , $P < 0.0001$; Ag vs. GEM, $P = 0.0005$; CFA vs. GEM, $P = 0.0133$), and (D) M-MDSCs (One-way ANOVA, Tukey's post-hoc test, n = 5 per group; CFA vs. Ag , $P = 0.3431$; Ag vs. GEM, $P = 0.1539$; CFA vs. GEM, $P = 0.8522$) in spleens from CFA-, Ag-, and GEM mice at 18 weeks of immunization

Data are representative of three independent experiments with similar results.

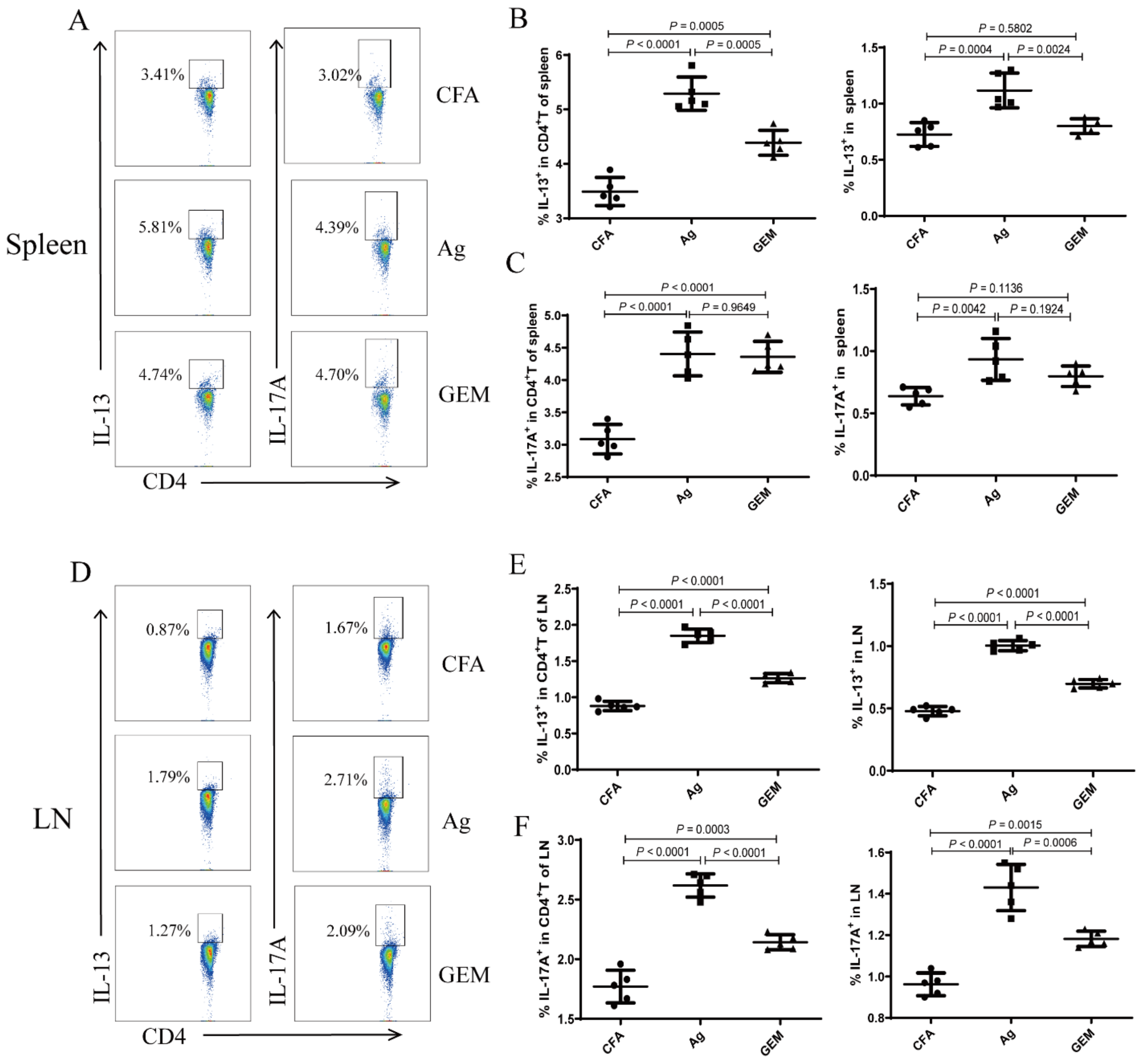

Supplementary Figure 3. The proportion of T helper 2 (Th2) and T helper 17 (Th17) cells decreased in the spleen and lymph nodes (LN) of mice treated with gemcitabine.

(A and D) Representative staining profiles of IL-13 and IL-17A in spleen (A) and lymph nodes (D) from Complete Freund's Adjuvant **(**CFA)-, primary membranous nephropathy (PMN) model (Ag)-, and gemcitabine-treated (GEM) mice at different immunization time points **(B and E)** Percentage of interleukin (IL)-13 in $CD4^+$ T cells (left panel) and peripheral blood mononuclear cells (PBMCs) (right panel) in the (B) spleens (One-way ANOVA, Tukey's post-hoc test, n = 5 per group; left panel: CFA vs. Ag , $P < 0.0001$; Ag vs. GEM, $P = 0.0005$; CFA vs. GEM, $P = 0.0005$; right panel: CFA vs. Ag , $P = 0.0004$; Ag vs. GEM, $P = 0.0024$; CFA vs. GEM, $P = 0.5802$) and (E) lymph nodes (One-way ANOVA, Tukey's post-hoc test, n = 5 per group; left panel: CFA vs. Ag , $P < 0.0001$; Ag vs. GEM, *P*

< 0.0001; CFA vs. GEM, $P < 0.0001$; right panel: CFA vs. Ag , $P < 0.0001$; Ag vs. GEM, $P < 0.0001$; CFA vs. GEM, $P < 0.0001$)from representative CFA-, Ag-, and GEM mice at 18 weeks of immunization **(C and F)** Percentage of IL-17A in $CD4^+$ T cells (left panel) and PBMCs (right panel) in the (C) spleen (One-way ANOVA, Tukey's post-hoc test, n = 5 per group; left panel: CFA vs. Ag , $P < 0.0001$; Ag vs. GEM, $P = 0.9649$; CFA vs. GEM, $P < 0.0001$; right panel: CFA vs. Ag , $P = 0.0042$; Ag vs. GEM, $P = 0.1924$; CFA vs. GEM, $P = 0.1136$) and (F) lymph nodes (One-way ANOVA, Tukey's post-hoc test, n = 5 per group; left panel: CFA vs. Ag , $P < 0.0001$; Ag vs. GEM, $P < 0.0001$; CFA vs. GEM, $P = 0.0003$; right panel: CFA vs. Ag , $P < 0.0001$; Ag vs. GEM, $P = 0.0006$; CFA vs. GEM, $P = 0.0015$) from representative CFA-, Ag-, and GEM mice at 18 weeks of immunization

The data represent three independent experiments.

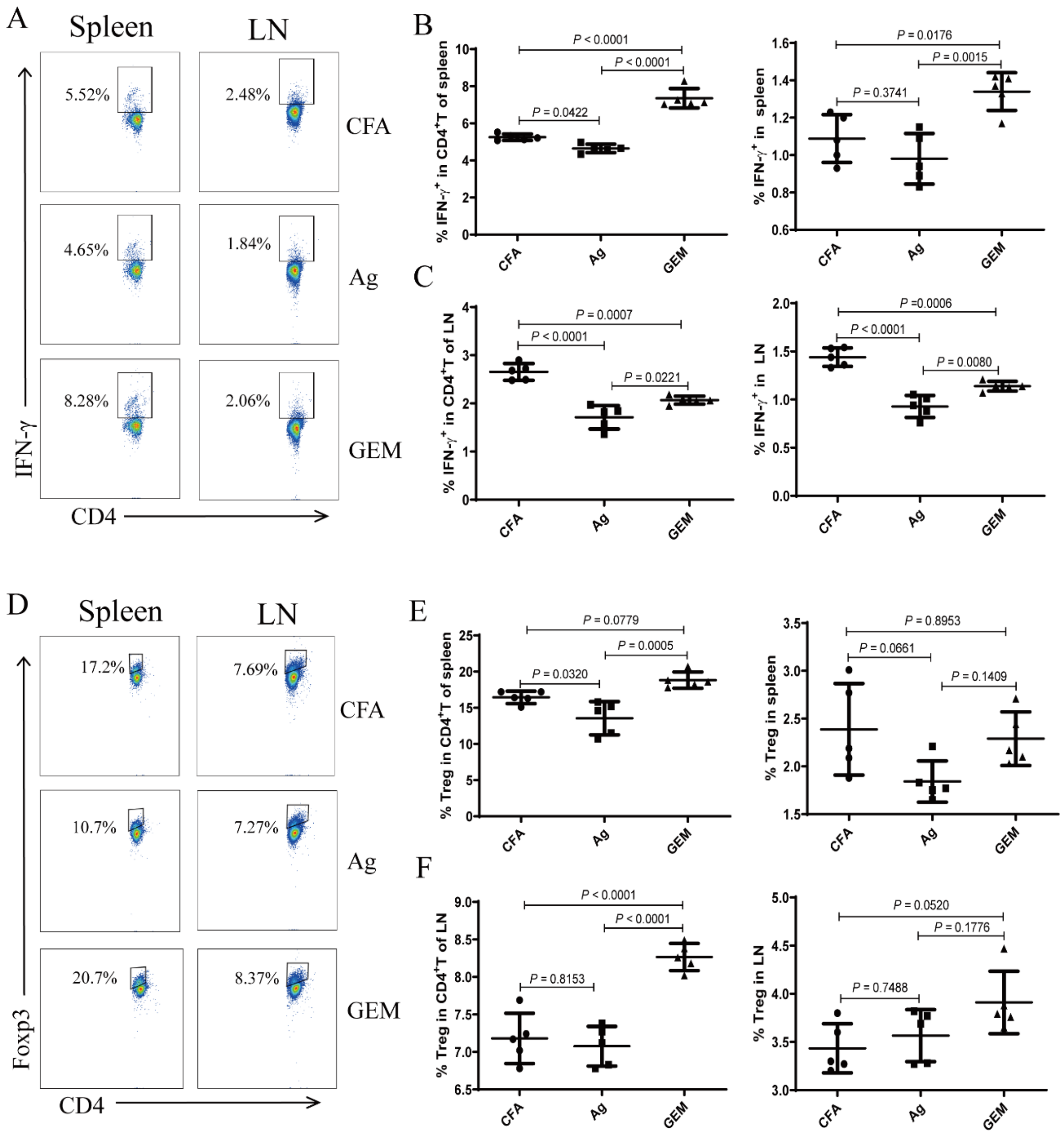

Supplementary Figure 4. The proportion of T helper 1 (Th1) and regulatory T (Treg) cells increased in the spleen and lymph nodes of mice treated with gemcitabine.

(A and D) Representative staining profiles of interferon (IFN)-γ (A) and Forkhead box P3(Foxp3) (D) in the spleen and lymph node from Complete Freund's Adjuvant **(**CFA)-, primary membranous nephropathy (PMN) model (Ag)-, and gemcitabine-treated (GEM) mice at different immunization time points **(B and C)** Percentage of IFN-γ in $CD4^{+}$ T (left panel) and peripheral blood mononuclear cells (PBMCs) (right panel) in the (B) spleen (One-way ANOVA, Tukey's post-hoc test, n = 5 per group; left panel: CFA vs. Ag , $P = 0.0422$; Ag vs. GEM, $P < 0.0001$; CFA vs. GEM, $P < 0.0001$; right panel: CFA vs. Ag , $P = 0.3741$; Ag vs. GEM, $P = 0.0015$; CFA vs. GEM, $P = 0.0176$) and (C) lymph nodes

(One-way ANOVA, Tukey's post-hoc test, n = 5 per group; left panel: CFA vs. Ag , $P < 0.0001$; Ag vs. GEM, $P = 0.0221$; CFA vs. GEM, $P = 0.0007$; right panel: CFA vs. Ag , $P < 0.0001$; Ag vs. GEM, $P = 0.0080$; CFA vs. GEM, $P = 0.0006$) from representative CFA-, Ag-, and GEM mice at 18 weeks of immunization **(E and F)** Percentage of Tregs in $CD4^+$ T cells (left panel) and PBMCs (right panel) in the (E) spleen (One-way ANOVA, Tukey's post-hoc test, n = 5 per group; left panel: CFA vs. Ag , $P = 0.0320$ Ag vs. GEM, $P = 0.0005$; CFA vs. GEM, $P = 0.0779$; right panel: CFA vs. Ag , $P = 0.0661$; Ag vs. GEM, $P = 0.1409$; CFA vs. GEM, $P = 0.8953$) and (F) lymph nodes (One-way ANOVA, Tukey's post-hoc test, n = 5 per group; left panel: CFA vs. Ag , $P = 0.8153$; Ag vs. GEM, $P < 0.0001$; CFA vs. GEM, $P < 0.0001$; right panel: CFA vs. Ag , $P = 0.7488$; Ag vs. GEM, $P = 0.1776$; CFA vs. GEM, $P = 0.0520$) from representative CFA-, Ag-, and GEM mice at 18 weeks of immunization

The data represent three independent experiments.

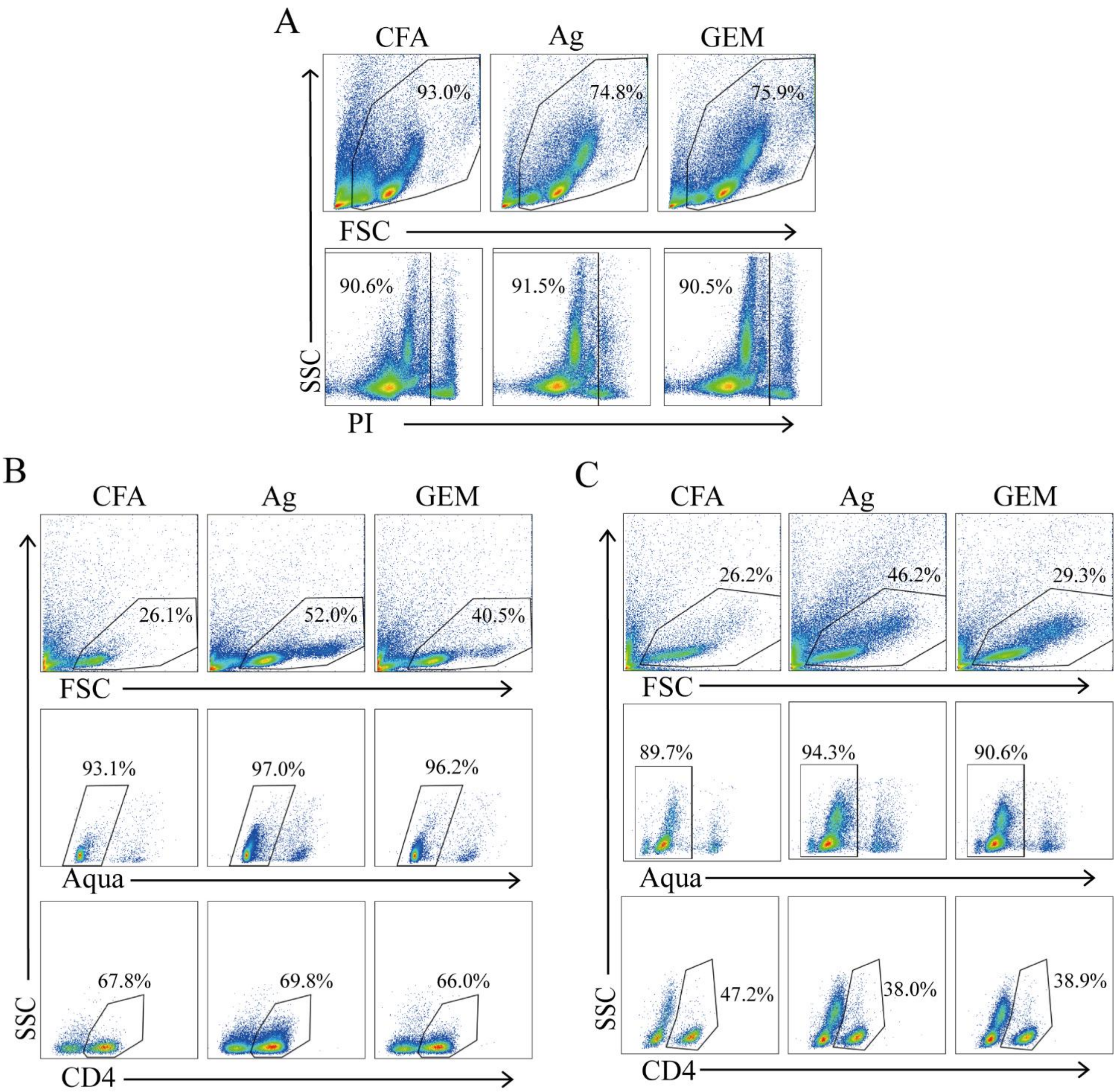

Supplementary Figure 5. Staining gating strategy profiles for detecting myeloid-derived suppressor cells (MDSCs), T helper 17 (Th17)/ T helper 2 (Th2)/ T helper 1(Th1) and regulatory T (Treg) cells by flow cytometry from representative Complete Freund's Adjuvant (CFA)-, primary membranous nephropathy (PMN) model (Ag)-, and gemcitabine-treated (GEM) mice

(A) Representative gating strategy profiles for detecting MDSCs to deplete dead cells with propidium iodide (PI) staining (SSC: Side Scatter; FSC: Forward Scatter) **(B and C)** Representative gating strategy profiles for detecting Th17/Th2/ Th1 (B) and Treg (C) cells to deplete dead cells with Aqua (LIVE/DEAD Fixable) staining

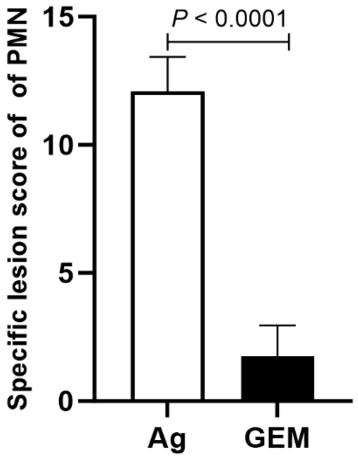

Supplementary Figure 6. Specific lesion score of primary membranous nephropathy (PMN) model (Ag) and gemcitabine-treated (GEM) mice

The 20 visual observations were randomly selected for each group, which were evaluated by 3 experienced pathologists based on light microscopy and electron microscopy, and the specific lesion score of PMN was calculated by adding up the scores according to the following indicators: (1) Degree of immune complex deposition and thickening of the basement membrane: Stage I: 1 point; Stage II: 2 points; Stage III: 3 points; Stage IV: 4 points (2) Intensity and extent of immunofluorescence: 0 points: no deposition; 1 point: focal (< 50% of glomeruli) weakly positive (+); 2 points: diffuse (> 50% of glomeruli) moderate intensity (++); 3 points: diffuse strong positive (+++) (3) Glomerular sclerosis (global / segmental): 0 points: no sclerosis; 1 point: sclerosis ratio < 25%; 2 points: 25% - 50%; 3 points: > 50% (4) Mesangial proliferation / matrix expansion: mild (only a few glomeruli with widened mesangial area) 1 point, moderate (dilated mesangial area in some glomeruli) 2 points, severe (significantly widened mesangial area in most glomeruli) 3 points (5) Podocyte injury: combined with swelling and vacuolar degeneration of podocytes under light microscopy, or fusion range of podocyte protrusions under electron microscopy (< 25% = 1 point, 25% - 50% = 2 points, > 50% = 3 points) (Mann-Whitney U test, $P < 0.0001$)
